# The Asbestos Ban in Korea from a Grassroots Perspective: Why Did It Occur?

**DOI:** 10.3390/ijerph15020198

**Published:** 2018-01-25

**Authors:** Yu-Ryong Yoon, Kyeong Min Kwak, Yeyong Choi, Kanwoo Youn, Jinwook Bahk, Dong-Mug Kang, Domyung Paek

**Affiliations:** 1 Department of Environmental Health, School of Public Health, Seoul National University, Seoul 08826, Korea; yyy001@hanmail.net (Y-R.Y.); ggm1981@snu.ac.kr (K.M.K.); 2 Department of Occupational and Environmental Medicine, Gachon University Gil Medical Center, Incheon 21565, Korea; 3 Asia Citizen’s Center for Environment and Health, Seoul 03184, Korea; choiyy@kfem.or.kr; 4 Department of Occupational and Environmental Medicine, Wonjin Green Hospital, Seoul 02228, Korea; dudunanum@hanmail.net; 5 Department of Public Health, Keimyung University, Daegu 42601, Korea; jwbahk@gmail.com; 6 Department of Occupational and Environmental Medicine, Pusan National University Yangsan Hospital, Yangsan 50612, Korea; kangdm@pusan.ac.kr; 7 Institute of Health and Environment, Seoul National University, Seoul 08826, Korea; paekdm@snu.ac.kr

**Keywords:** asbestos ban, Korea, narrative analysis, health-oriented cultural change, health and safety stage, grassroots

## Abstract

In 2009, asbestos was finally banned in Korea, about 70 years after the first opening of asbestos mines under Japanese control. After having presented the history of asbestos industry, together with its regulations and health effects over time, we constructed narrative analyses of how the asbestos issue under the prevailing risk system was managed by whom and for what purpose, to provide context for the change. We could identify five different phases: laissez-faire, politico-technical, economic–managerial, health-oriented cultural, and human rights-based post-cultural risk systems. The changes leading to the asbestos ban evolved over different phases, and each phase change was necessary to reach the final ban, in that, without resolving the previous issues by examining different categories of potential alternatives, either the final ban was not possible or, even if instituted, could not be sustained. An asbestos ban could be introduced when all the alternatives to these issues, including legitimate political windows, economic rationalizations, health risk protections, and human rights sensitivities, were available. We think the alternatives that we had were not in perfect shape, but in more or less loosely connected forms, and hence we had to know how to build solidarities between different stakeholders to compensate for the imperfections.

## 1. Introduction

The manufacturing, use, and import of all asbestos products as well as raw asbestos were banned in Korea in 2009, after two years of probation from 2007. This was about 50 years after the first manufacturing of asbestos cement products, and about 70 years after the first opening of asbestos mines under Japanese control. The first diagnosis of malignant mesothelioma in relation to occupational exposure was made in 1993, and even after the official recognition of the causality, it took more than 15 years to implement the ban.

Here we present the history of the asbestos industry in Korea, especially in the aspects of health and safety, and describe the accompanying social and administrative changes over the period. We have focused on the operation of industries directly handling raw asbestos, asbestos mining, and asbestos product manufacturing, and examined the time trends of asbestos consumption together with the import and export of asbestos products. Next we have examined the changes in asbestos regulations, together with the exposure levels of air-borne asbestos in major industries over the period. As for the health and safety effects themselves, we have examined the officially recognized cases of asbestos-related occupational diseases, and annual mesothelioma cases in the general population. After the banning of asbestos, environmental victims of asbestos exposure began to be compensated, and we have also summarized the current status of environmental asbestos problems.

In the analysis of why such changes occurred, we first identified all the relevant issues and participating stakeholders that contributed to the changes over the periods, and then grouped those issues and involved actors into series of distinctive phases. In describing each phase, we have constructed narrative analyses of how the issues were managed under the prevailing risk system—by whom and for what purpose—to provide context for the change. Here we conceptualized the risk management system to predict, execute (do), and then check the risk to health and safety of key system stakeholders so that the larger system harboring the subsystem of risk management can be maintained for the foreseeable future. When the predictions of the risk management system had failed, as evidenced by the appearance of new health and safety issues, we tried to identify what alternatives were sought for the emerging problems, and why risk management system had changed from one phase to another.

In Korea, at the beginning, there were no issues and no major stakeholders, and so risk management was in the state of ‘laissez-faire’. After this initial ‘laissez-faire’ state, a series of issues emerged, prompting changes in technical, managerial, and cultural aspects of risk management systems and then for the consolidation of post-cultural changes in health and safety status. We noticed that, in tackling the problems surrounding these issues, alternative measures were formulated mainly by the stakeholders of political, economic, health-oriented, and human rights-based interventions. Based on these two features, the nature of major issues and the sort of major stakeholders, we could identify five different phases: laissez-faire, politico-technical, economic-managerial, health-oriented cultural, and human rights-based post-cultural risk systems.

## 2. Asbestos Problems and Management

### 2.1. The Asbestos Industry and Asbestos Usage

During the 1930s, asbestos was first mined for use in shipbuilding for the Japanese navy [1]. After the end of World War II in 1945, all mining stopped, and only opened again in 1950s after the Korean War for the slate industry in the newly independent Korea. During the further industrialization in the 1960s–1970s, asbestos mining continued, especially due to the increased demand of asbestos slate for renovation of traditional roofs in rural areas. However, because of the cost, quantity, and quality, domestic production of asbestos was abandoned and replaced with cheaper imports, especially from Canada, and the last asbestos mine was closed in 1983. Later, in the 2000s, the areas around the closed mines and the roads to the old railroad station facilities for transport were found to be contaminated with the remaining asbestos waste from previous mining activities [2].

Manufacturing of construction materials, friction products, textiles, and gaskets were the main industries of raw asbestos use, and with the growth of automobile and other heavy industries, the demand for asbestos products increased up to the early 1990s. As indicated above, the manufacturing of asbestos slates and other construction materials started in the 1950s. The asbestos textile industry started in the late 1960s, and in 1971, just before the implementation of the Japanese Industrial Safety and Health Law, Japanese asbestos textile firms moved their machinery under the guise of foreign investments [3]. Later, German firms also joined this transfer of dangerous industries in the midst of rapid and rather ill supervised industrialization [4]. Eventually, asbestos textile plants moved to Indonesia and China in the early 1990s [3]. Meanwhile, various industries of ship building, construction, plumbing and heating, auto mechanic, and other machine operations had used asbestos products widely up to the early 2000s.

**Figure 1:**
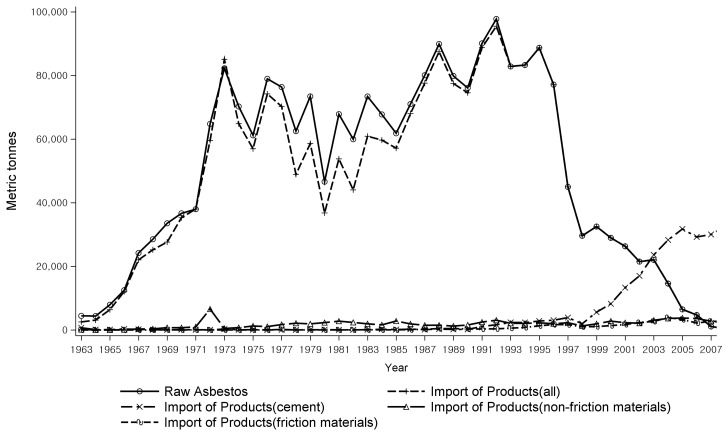
The amount of raw asbestos and asbestos products imported to Korea during 1963–2009.

As raw asbestos imports and asbestos products manufacturing decreased in Korea from the late 1990s, the import of manufactured asbestos products increased instead until 2006 (Figure 1) [5]. During the last 10 years before the asbestos ban, asbestos substitute industries had expanded, and almost no industries using raw asbestos were left in Korea at the time of the asbestos ban.

### 2.2. Asbestos Regulation and Issues

Regulation of asbestos in Korea began only when the Industrial Safety and Health Law was enacted in 1981. Previous to that, no specific regulations existed. However, even though the law had specified that the manufacturers of asbestos products should register and get government authorization, no routine measurements of air-borne asbestos was made until the standard on workplace environment measurement method was issued in 1987 (Ministerial Notice 86–46).

**Figure 2:**
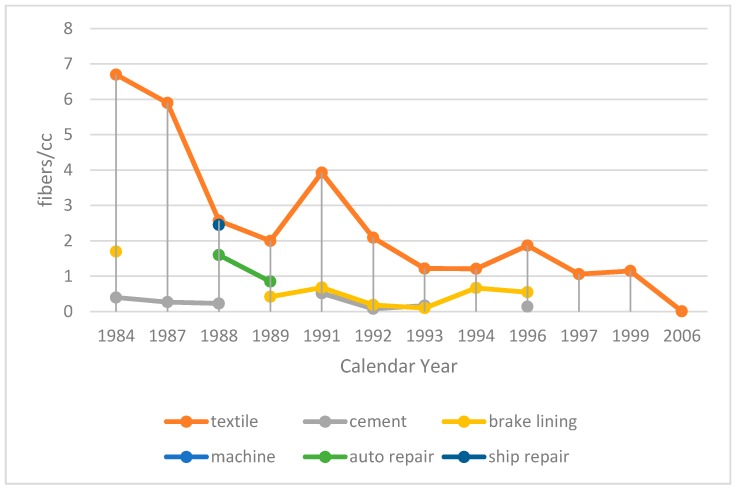
The level of asbestos air monitoring for different industries from 1984 to 2006.

In 1986, the occupational exposure limit to asbestos was set at 2 fibers/cc, and then lowered to 0.1 fibers/cc in 2003. The level of asbestos at the workplace in the 1987 survey, however, was much higher than the exposure limit, especially in textile industries (Figure 2) [1]. Later, in the 1990s, the exposure level began to decrease to under 1 fiber/cc with repeated measurements [6].

Crocidolite and amosite were banned in 1997, but no use was found for them at the time of banning. Asbestiform fibers of tremolite, anthophyllite, and actinolite were banned in 2003, and chrysotile was finally banned in 2009. After banning, controversy erupted about the definition of asbestos-containing products. The Ministry of Labor had changed the lower limit for asbestos-containing products from 1% to 0.1% of asbestos by weight in 2007. However, the definition used by the Ministry of the Environment was still 1% of asbestos by weight to be considered as asbestos-containing in 2008, based on the X-ray diffraction detection limit of 0.5%. In 2009, the Ministry of the Environment widened the definition to 0.1% for asbestos-containing talc.

### 2.3. Asbestos Problems and Predictions

**Figure 3:**
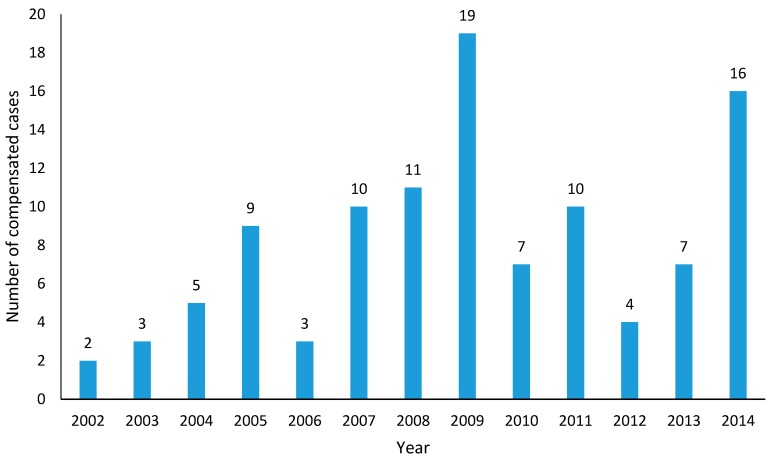
The number of cases compensated for asbestos-related occupational diseases in Korea between 2002 and 2014.

The first case of asbestos related mesothelioma was diagnosed in a female worker in 1993, and soon recognized as work-related in 1994, based on a single history of 19 years of work at an asbestos textile factory [1]. Cases of asbestosis and asbestos-related lung cancer were reported over the ensuing years [7,8,9]. However, it took another eight years to have the second compensation officially accepted in 2002, and still the number of cases compensated for asbestos-related occupational diseases remains fewer than 20 per year [10] (Figure 3). When compared to European countries [11], the number (fewer than 10 cases/year) and proportion (less than 50%) of recognized lung cancer from asbestos exposure remains relatively small [12].

As of 2013, the incidence of malignant mesothelioma in the general population was 2.8 per million person-years for men and 1.55 per million person-years for women, with a sex ratio of 1.9:1 [13]. Compared to other developed countries, the incidence of malignant mesothelioma and also the sex ratio are still relatively low. This is estimated to increase continuously up to 20 more years based on the age cohort and period models, but the change in age structures rather than the age standardized incidence itself is expected to contribute to the major increase in the predicted crude incidence over the period [13].

Environmental victims of lung cancer or asbestosis with an adequate residence history, or malignant mesotheliomas of any location, have been compensated under the Asbestos Injury Relief Act from 2011. From 2014, diffuse pleural thickening was added to the compensable list. Up to December 2017, a total of 2801 cases were compensated, including 914 malignant mesotheliomas, 409 lung cancers, 1474 asbestosis, and four diffuse pleural thickenings [14]. About half of all compensated cases had occupational history of asbestos exposure, but could not be covered by Industrial Accident Compensation Insurance because of the eligibility criteria of exposure and the differences in the diagnostic technique of pneumoconiosis (simple X-ray for occupational compensation versus CT for environmental relief) [15]. Even considering the requirement of residence histories, the number of compensated lung cancer cases is relatively small compared to that of mesothelioma or asbestosis.

## 3. Phases of Asbestos Problems Unfolding over the Period

In the analysis of the context of the major changes, based on the discussion of the salient issues over the periods and the narratives about what, how, who, and why, we have divided the whole period into five different phases.

After the initial phase of ‘laissez-faire’, the risk of asbestos was managed first by administrative enforcements of technical measures to employers in the form of law enactment. This type of enforcement was a major political decision, and major stakeholders at this ‘politico-technical’ stage were politicians backed by technocrats and professionals. At this stage, risk assessments in the form of workplace exposure measurement based on code-based approaches including national legislations were dominant, and the problems of risk were constructed based on data gathering only without any further analysis.

After instituting employers’ duty to deploy technical measures, however, the logistics and feasibility of the mandated techniques in terms of costs and quality became a major issue. The major stakeholders in the next ‘economic–managerial’ stage were those formulating alternatives for the economy and management. Risk management based on labor negotiations and corporate policy, such as risk allowance to exposed workers, became dominant, and the problems were constructed as the calculated risk based on processed information, such as exceedance rate over exposure limits, but still not validated, nor verified.

When the economics of risk-taking by relevant parties including employers and unions were proved to be unsafe by the emergence of victims, the need for a cultural change in risk management became self-evident. The alternatives, including the asbestos ban, were formulated mainly by stakeholders oriented to health issues. In this ‘health-oriented cultural change’ stage, risk communication based on actual experience became essential, and the problems were constructed based on independently validated knowledge about the nature of health and safety risks.

Finally, various needs for the consolidation of post-cultural changes in health and safety measures including compensation, reduction, and primary prevention of risks became necessary, as the lessons for the future had to be drawn from all different angles surrounding the actual experiences. In particular, precautionary measures in the form of prevention were found to be dysfunctional without human rights-based approaches, and the role of those advocating human rights became essential. In this ‘human rights-based post-cultural’ stage, continuous cycle of risk assessment, management, and communication became a prerequisite for most stakeholders, and the problems were constructed based on embodied wisdom, especially that of the victims.

The following table gives an overview of five different stages of health and safety change (Table 1).

### 3.1. Laissez-Faire Period (Up to 1980)

Before 1980, asbestos was not an issue in Korea. Except for very few medical professionals with contacts in foreign countries, no particular stakeholders paid attention to the problems of asbestos.

### 3.2. Politico-Technical Period (1981–1987)

In 1981, the Industrial Safety and Health Law was introduced in the middle of the transition to another military regime in Korea. At the time, no particular health and safety issues were raised beforehand, and health and safety measures were introduced rather as a political maneuver. In a similar vein, the Pneumoconiosis Act was enacted in 1984. However, unlike in Japan, only miners of certain minerals were covered, leaving all asbestos workers out of reach, as the Act was enacted due to the riots of coal miners in 1980. In this political phase, health and safety issues were used as a way to legitimize the regime. International standards, such as the Industrial Safety and Health Law, were adopted with an emphasis only on the technical aspects of health and safety, thereby medicalizing the problems, while maintaining the status quo of management–labor relations and ignoring any potential human rights issues.

In this phase, technocrats and professionals played a major role, but no unions, civil groups, or victims had their voices heard. Risky and expensive mining activities were curbed, and asbestos mines were closed in the middle of the period.

Legitimate political windows, not just political manipulations by politicians, were, however, not available until the democratic process could enable marginalized health and safety victims to exercise their rights to political freedom. In 1987, the autocratic constitution was revised by the Korean people’s June Democracy Movement, and a foundation was laid for the opening of legitimate political windows when necessary. Democratization of the political process finally put an end to this politico-technical phase.

### 3.3. Economic–Managerial Period (1988–1994)

After the democratization of politics, an avalanche of health and safety issues was brought to the political arena’s attention, and training of new occupational medicine, nurse, and hygiene specialists and the restructuring of occupational health and safety institutions were at the center of the discussion.

Growing attention to economics led to the closing of the less competitive asbestos textile plants and the closed plants eventually moved to countries with no regulations in the early 1990s [3]. During this period globalization expanded, especially in the economic sphere, and auto industries had to adopt asbestos-free brake linings for their exported cars, while shipbuilding industries had to avoid asbestos products for new ships. In this sense, the U.S. EPA ‘Asbestos Ban and Phase-Out Rule’ in 1989, even though it was later overturned by the Court in 1991, was important for the trading partner countries. As economic activities were globalized, the alternatives of health and safety, including technologies, managements, trades, legal standards for trainings and educations, etc., were also globalized, even to a limited extent, and the asbestos substitute industry grew further during this period.

In this period, government officials, professionals, and unions played major roles, and between them, the issues of health and safety often morphed into hazard incentives or job security. For example, auto assembly workers working on mufflers with asbestos gaskets once received extra income as a hazard incentive through negotiations. However, this lenient attitude was soon shaken by eyewitness accounts of asbestos problems, first in the form of the totally unexpected but undeniable diagnosed case in 1993, and then with the further accumulation of epidemiologic evidence, and worries about potential asbestos health risk finally put an end to the managerial–economic phase [7].

### 3.4. Health-Oriented—Cultural Change Period (1995–2009)

As the perception of the health risk was awakened by the appearance of concrete medical problems, the economic–managerial phase was transformed into a health-oriented cultural change phase. This awakening was assisted by two interconnected movements in Korea, first the outreach of activists and professionals, and second by the gatherings of the victims.

International developments, including the foundation of the International Ban Asbestos Secretariat (IBAS) in 1999 and decision of the World Trade Organization (WTO) on the asbestos trade in 2001, were particularly important for activists and professionals in sharing and disseminating ideas [16]. A series of gatherings of activists and victims in Osasco, Brazil (2000), Tokyo, Japan (2004), and Bangkok, Thailand (2006) provided momentum for sharing experiences and strategies towards the asbestos ban in Korea [17]. This led to the investigation of subway workers (2004) [18] and residents near old asbestos facilities (2007) [19,20], even including a series of visits to Indonesia to study the transferred asbestos textile factories from Busan, Korea [3].

The first recognition of occupational cancer in 1994 stirred up the victims. In particular, victims and family members of the same asbestos textile factory in Busan became conscious of the risks, and the first lawsuit for the recognition of asbestos-related disease by a co-worker of the first victim was filed in 2006. A campaign was organized in 2007 to find the whereabouts of the previous workers to share experiences and information [21]. A similar gathering of victims was formed in Chungnam Province, Korea, among residents near old asbestos mines after an environmental study in 2007 [22].

After the Kubota shock of Japan in 2005 [23], exchanges of information about asbestos problems between Korea and Japan were made on various levels, including meetings of professionals between members of Japan Federation of Democratic Medical Institutions and the Korean Association of Physicians for Humanism in 2006 (Seoul, Korea), and visits of victims and family members of Korea to the Kubota plant in Amagasaki, Japan in 2007.

These activities led to the formation of the Ban Asbestos Network Korea (BANKO) in 2008. BANKO was an umbrella group of victims and families, environmental movements, unions, professionals, and health and safety activists, working sporadically with politicians. A series of concerns about the environment was raised by BANKO with the reports of asbestos contamination in previously unsuspected situations, such as contamination of talc baby powders (2009) and naturally occurring asbestos in playgrounds [24,25].

Based on these activities, victims and NGOs became regular stakeholders in setting policy on asbestos. With these looming possibilities of environmental risks to health, an asbestos ban in 2009 was accepted as a legitimate health-oriented solution, and the Ministry of Environment became the lead department for asbestos policy in Korea. However, to fulfill the objectives of an asbestos ban, precautionary measures became necessary even when concrete evidence was lacking. The discussion on precautionary management had shifted from a more or less health-oriented one to a human rights-based one. This change in the basis of risk perception led to the end of the health-oriented cultural change phase of risk management.

### 3.5. Human Rights-Based—Post-Cultural Period (2010–)

Even after the ban, issues of asbestos periodically erupted. First, while some basic compensation programs, even though somewhat dysfunctional due to the inherent deficiencies of the Pneumoconiosis Act, as mentioned above, were available for workers with asbestos-related diseases, no such measures were present for environmental victims. To reveal the extent of environmental problems, activists and professionals emphasized that compensation should be provided to the environmental victims identified in studies near old mines or old asbestos product manufacturing facilities [26]. The Asbestos Injury Relief Act was enacted in 2011 following a Japanese model.

Second, in addition to the old asbestos contamination sites from the 1960s and 1970s, relatively recent installments from the 1980s and 1990s were widespread, and even fresh imports after the ban were identified laden with asbestos products such as asbestos bicycle brakes. Together with these, removal of asbestos installments from public buildings and facilities, large-scale renovation projects in inner city areas, remediation of naturally occurring asbestos installed in public areas such as baseball grounds, school grounds, bicycle paths, golf courses, and parks presented a series of questions about the safety of the management policy and its practices, especially the sloppy attitudes of government officers. These discussions led to the enactment of the Asbestos Safety Management Act in 2012.

Specifications in the Asbestos Safety Management Act include the mapping of asbestos installments, the right to know of concerned parties, including not only workers but also the general public, the establishment of a code of practice for the removal process and qualification procedures to provide removal services, and the safe handling and disposal of asbestos waste. All the stakeholders, including NGOs and victims, had to ensure that these specifications are met.

## 4. Lessons Learned

The changes leading to the asbestos ban evolved over different phases, and each phase change was necessary to reach the final ban, in that, without resolving the previous issues by examining different categories of potential alternatives, either the final ban was not possible or, even if instituted, could not be sustained. Countries may have instituted the ban purely based on political or administrative needs, but we know some soon reversed their position when other conditions, such as economic feasibility or a sound social basis for health protection, were not met [27].

First, the chaotic phase had ended and the political phase began. It was the political needs of autocratic leaders to legitimize the regime that could have brought the international health and safety standard into law. Only after the democratization of the political process, however, did political discussions expand to deal with wider issues of social importance. Later this change laid the foundation for the opening of political windows by civil society and victim groups when necessary.

Once the politico-technical issues were resolved, all sorts of economic alternatives were sought for the more rationalized economy in the economic–managerial phase. Even with the economy-oriented institutional and managerial reforms, such as the creation of new specialties and the transfer of dangerous industrial processes abroad, the accumulated evidence of management risks in the form of health became evident with the appearance of victims, and the activities of economic rationalization had to give way to health-oriented cultural changes. In fact, economic rationalization was there from the beginning, even under the limits of political constraints, and the Japanese asbestos textile firm that moved to Korea during its ‘laissez-faire’ stage, just before the Japanese Industrial Safety and Health Law of 1972, had withdrawn its investment from Korea when Korea was ready to enact its own Industrial Safety and Health Law in 1980. Upon examining the trends in asbestos consumption more closely, one notices that the total amount plateaued in the 1980s in Korea (Figure 1), during which time the asbestos substitute industry for brake linings and ship buildings had already started. Based on this, the relative shrinkage of asbestos consumption when compared to the overall industrial growth in Korea should have started much earlier than the actual shrinkage in the 1990s. Wider economic alternatives in the globalized economy with examples of asbestos substitute industries from trade partners further contributed to the decline of the asbestos industry during the economic–managerial phase and later.

**Figure 4:**
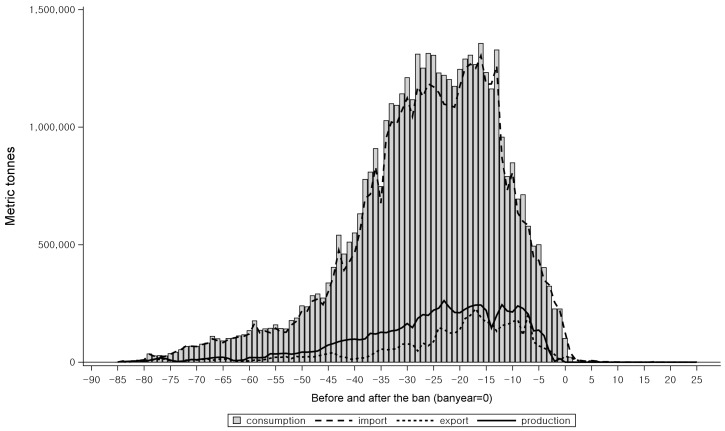
Crude asbestos production, import, export, and consumption among high-income banned countries from 1920 to 2011. Different calendar years were adjusted to year 0 for all the countries with an asbestos ban.

**Figure 5:**
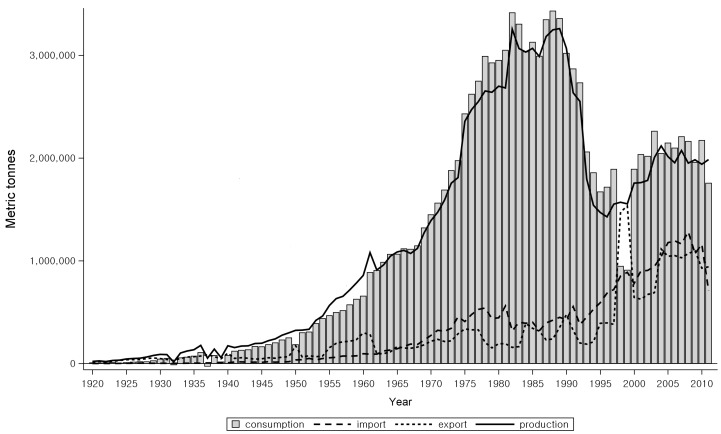
Crude asbestos production, import, export, and consumption among middle- and low-income no-ban countries from 1920 to 2011.

When we examined the pre-ban status of asbestos industry in those countries identified by IBAS to have instituted an asbestos ban, the usage of raw asbestos had dwindled down to an almost negligent level before they instituted the ban in all the countries (Figure 4) [5]. Meanwhile, in those countries without a ban, as identified by IBAS, the total amount of asbestos consumed decreased but had not yet dropped to nil over the examined periods (Figure 5) [5]. When Korea banned crocidolite and amosite in 1997, and asbestiform tremolite, anthophyllite, and actinolite in 2003, there were no industries that used those minerals. In this regard, we think economic rationalization of the asbestos industry, whether as a result of change in health risk perception or as a result of economic risk appraisal, is necessary for the implementation of the final ban.

With these changes in the background, after transitioning from the politico-technical and then the economic–managerial phase, an asbestos ban could be brought about by cultural change with the increasing evidence of asbestos-related health problems. Here, health risks were perceived as more than just as a potential threat, based on neighborhood experiences such as the 2001 WTO decision in the asbestos dispute between Canada and France, and the 2005 Kubota shock in Japan. It should be noted that environmental health threats were perceived as pivotal in the discussion of a ban in the mass media.

Even after the asbestos ban, the ban was not effective without consideration of human rights, especially for children and the following generations who will bear the lasting burden of asbestos. In stressing human rights, it was also important to avoid too much medicalization of the problem, because medical alternatives could not be the final answer to the root cause of the problem, as evidenced by the pneumoconiosis program in Korea. In fact, we found that blind medicalization without a balanced crosscheck between politics, economy, health, and human rights, can present serious obstacles to problem-solving in that institutions with occupational disease compensation in Korea once required biopsies to diagnose asbestosis victims.

As for the stage transitions in Korea, we think we could observe the aforementioned sequence of changes because easier alternatives had to be adopted in earlier stages while more difficult ones were reserved for later stages. Because of this ease of change, we think that the technical measures, as advocated by technocrats and professionals, were adopted in the first place by the political decisions. The economic rationalization, the health protection, and the human rights-sensitive changes were much more difficult to achieve, and because of these difficulties they were deferred to later stages. In short, we think that addressing the easier dimensions of health and safety issues led to a search for alternative solutions for the remaining but more difficult problems, and this search eventually resulted in transitions from one stage to another.

The final point to stress is that, as the precautionary principles could only be applied based on a human rights approach, we found that solving the asbestos problem was a powerful lesson with good examples for other health and safety problems. In fact, we saw some other spin-offs of carcinogen awareness, so that the carcinogen-zero movement of consumer groups and environmental NGOs started in 2009, and the occupational cancer recognition movement by the major trade unions was initiated in 2010 through mass filing for compensation of occupational cancer.

## 5. Conclusions

In retrospect, all the changes were necessary for the ban [27]. In order to achieve the asbestos ban, first, legitimate political windows were necessary. Democratization of the political process freed the social discussions from political meddling, and later provided an opportunity for the opening of political windows for the victims. If the economic interests of the industry were tied to political power, we think the changes might have been very difficult, if not impossible.

Democratization also made the economic rationalization possible, together with wide-ranging economic alternatives. Some of the economic alternatives included asbestos substitute industries and even asbestos removal industries. In fact, after the asbestos ban, those who had imported raw asbestos turned to asbestos removal and protection services as they had hands-on experience about where asbestos was installed.

In the health-oriented cultural change phase, health science was pivotal in changing the risk perception culture, and later the processes and experiences of health studies, however rudimentary or low level they might be from the perspective of developed countries, were fundamental for building problem-solving procedures with medical alternatives including proper medical diagnosis and management.

Finally, we needed sensitivities of human rights in health to achieve the ban, and this was especially boosted with environmental health. The most intense and obvious problems lay in the occupational field, but we needed a broader approach of human rights for all—including socially disadvantaged occupational groups, children, aboriginals, and migrating workers.

An asbestos ban could be introduced when all the alternatives to these issues, including legitimate political windows, economic rationalizations, health risk protections, and human rights sensitivities were available. We think the alternatives that we had were not in perfect shape, but in a more or less loosely connected form, and hence we had to know how to build solidarities between different stakeholders to compensate for the imperfections.

## Figures and Tables

**Table 1:** Stages of health and safety changes based on narrative analysis.

Stages		Laissez-Faire	Technical	Managerial	Cultural	Post-Cultural
When		Up to 1980	1981–1987	1988–1994	1995–2009	2010–
Why (Objective)		Self-Interest Based	Politics Based	Economy Based	Health Based	Human Rights Based
Who (Key Role Players)		No Body	Government Employers	Professionals Unions	Victims NGOs	Everybody
What	Content	Self-care	Input Dominant	Process Dominant	Output Dominant	Input to Output
	Risk Handling	Innate Heuristics	Assessment	Management	Communication	Continuous Cycle
How	Politics	None	National Legislation	Corporate Policy	Court Cases	Open Mass Media
	Enforcement	Self-Discipline	Code Based	Labor Based	System Based	Precautionary Way
Problem Construct Level		None	Data	Information	Knowledge	Wisdom
